# Anti-Hu Antibody Associated Paraneoplastic Cerebellar Degeneration in Head and Neck Cancer

**DOI:** 10.1186/s12885-015-2020-4

**Published:** 2015-12-22

**Authors:** Florian Huemer, Thomas Melchardt, Wolfgang Tränkenschuh, Daniel Neureiter, Gerhard Moser, Teresa Magnes, Lukas Weiss, Alexander Schlattau, Clemens Hufnagl, Gerda Ricken, Romana Höftberger, Richard Greil, Alexander Egle

**Affiliations:** 1 Third Medical Department with Hematology, Medical Oncology, Hemostaseology, Infectious Diseases and Rheumatology, Oncologic Center, Salzburg Cancer Research Institute with Laboratory of Immunological and Molecular Cancer Research and Center for Clinical Cancer and Immunology Trials, Paracelsus Medical University Salzburg, Muellner Hauptstrasse 48, 5020 Salzburg, Austria; 2 Institute of Pathology, Paracelsus Medical University Salzburg, Salzburg, Austria; 3 Department of Otorhinolaryngology, Paracelsus Medical University Salzburg, Salzburg, Austria; 4 Department of Radiology, Paracelsus Medical University Salzburg, Salzburg, Austria; 5 Institute of Neurology, Medical University of Vienna, Vienna, Austria

**Keywords:** Anti-Hu antibody, Cerebellar ataxia, Paraneoplastic syndrome, Head & neck, Spindle cell carcinoma

## Abstract

**Background:**

Paraneoplastic syndromes are most frequently associated with small cell lung carcinoma, hematologic and gynecologic malignancies while reports in head and neck cancer are rare.

**Case presentation:**

We present the case of a 60-year old female patient who developed paraneoplastic cerebellar degeneration upon locoregional recurrence of a poorly differentiated spindle cell carcinoma of the nasal cavity and paranasal sinus. The neurological symptoms, especially ataxia, stabilized after resection of tumor recurrence and concomitant chemoradiotherapy whereas anti-Hu-antibodies remained positive. Despite the unfavorable prognosis of paraneoplastic neurological disorders associated with onconeural antibodies, the patient achieved long-standing stabilization of neurological symptoms.

**Conclusion:**

We report the first patient with anti-Hu antibodies and paraneoplastic cerebellar degeneration associated with a spindle cell carcinoma of the head and neck. We recommend that evaluation of neurological symptoms in patients with this tumor entity should also include paraneoplastic syndromes as differential diagnoses and suggest early extensive screening for onconeural antibodies.

## Background

Paraneoplastic syndromes are described as a heterogeneous group of disorders in the presence of a tumor with symptoms that do not originate from growth of cancer, metastatic spread, or infectious, toxic and metabolic effects associated with the underlying malignancy [1].

Depending on the affected organ system, endocrine, hematological, dermatological, rheumatological as well as neurological paraneoplastic syndromes have been described. For paraneoplastic syndromes, an association with various types of cancer – including small cell lung cancer (SCLC), thymoma, Hodgkin- and Non-Hodgkin-lymphoma and gynecological malignancies – has been demonstrated [2, 3].

Patients with primary cancer of head and neck rarely present with paraneoplastic syndromes, most of them are associated with squamous cell carcinomas [4, 5]. Among paraneoplastic neurological syndromes (PNS) in patients with head and neck cancer, only a few cases with paraneoplastic cerebellar degeneration (PCD) have been described so far [6, 7]. PCD is characterized by vertigo, nystagmus and rapidly progressing cerebellar ataxia with a poor median survival [3].

Diagnostic criteria recommended by the PNS Euronetwork help to distinguish “definite” from “possible” PNS depending on the presence of classical or non-classical neurological syndromes, presence of distinct onconeural antibodies, presence of a tumor, and improvement of symptoms after cancer therapy [8]. Treatment of cancer is still the main therapy of paraneoplastic neurological phenomena although development as well as persistence of PNS in remission have been described [9].

We report the first patient with a spindle cell carcinoma of the nasal cavity and paranasal sinus presenting PCD associated with anti-Hu antibodies and describe the clinical presentation as well as diagnostic work-up. Based on our findings, we suggest to screen neurologically symptomatic patients with head and neck tumors for a paraneoplastic syndrome.

## Case presentation

In November 2008, an otherwise healthy 60-year old Caucasian female presented at the department of otorhinolaryngology due to right-sided limited nasal breathing accompanied by bloody nasal discharge. Aside from tonsillectomy and partial dentures, her past medical history, including alcohol and nicotine, was unremarkable.

**Fig. 1:**
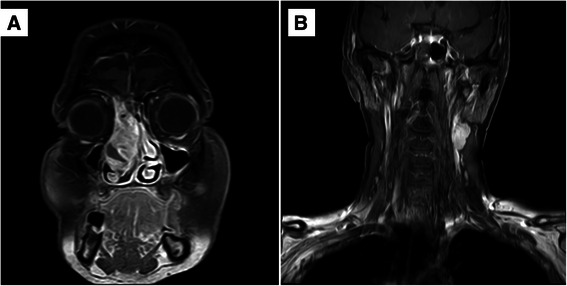
Magnetic Resonance Imaging of the Primary Tumor and Lymph Node Metastases. November 2008, coronal MRI with contrast of the head showing the primary tumor in the right main nasal meatus and ethmoid sinus (**a**). December 2009, coronal MRI with contrast of the head and neck demonstrating two spherical, enlarged cervical lymph nodes on the contralateral side (**b**)

**Fig. 2:**
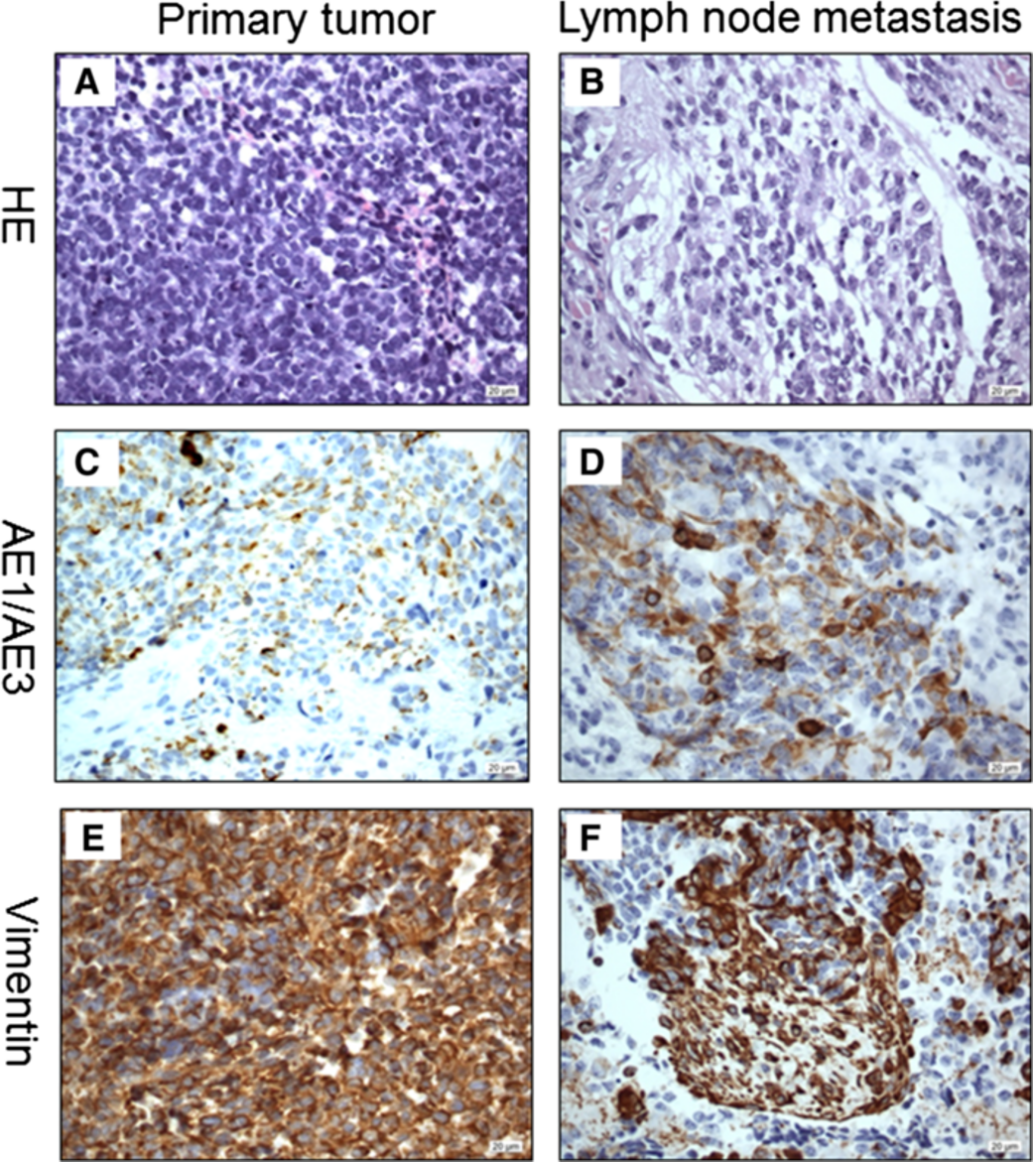
Histology and Immunohistochemistry of the Primary Tumor and Lymph Node Metastasis. Hematoxylin and Eosin (HE) stain of the primary tumor (**a**) and metastasis (**b**) shows the biphasic morphology of the spindle cell carcinoma. Immunohistochemical staining with AE1/AE3 demonstrates the epithelial elements of the primary tumor (**c**) and the cervical lymph node metastasis (**d**) whereas vimentin, a marker of epithelial-to-mesenchymal transition, spares these parts (**e**, **f**)

Examination with nasal specula showed a tumor covered with bloody secretions, completely occupying the right nasal meatus in the absence of cervical lymphadenopathy assessed by palpation. Magnetic resonance imaging (MRI) revealed nasal septal deviation due to a 5 × 2.2 × 4.5 cm measuring mass located in the right nasal meatus and right ethmoid sinus, highly suspicious of malignant origin (Fig. 1a). The tumor was removed by functional endoscopic sinus surgery. Based on histological and immunohistochemical results, showing epithelial as well as mesenchymal differentiation (Fig. 2), and absence of molecular translocation t(X,18), the diagnosis of a spindle cell carcinoma, formerly called carcinosarcoma, was made (cT3 N0 M0).

Following this diagnosis, 18F-fluorodeoxyglucose positron emission tomography/computed tomography (18F-FDG PET/CT) imaging showed a solitary 1 × 1.3 cm residual lesion in the right nasal meatus which necessitated surgical re-resection followed by adjuvant radiotherapy without adjuvant chemotherapy, leading to a cumulative radiation dose of 61.1 gray (Gy) in the tumor bed.

At follow-up in December 2009, a solitary firm and immobile mass beneath the left jaw angle was palpated on physical examination. Two spherical 1.5 cm measuring lymph nodes were detected by MRI (Fig. 1b) and intense tracer uptake was demonstrated by 18F-FDG PET/CT imaging.

Prior to the planned neck dissection, in January 2010, the previously asymptomatic patient complained about progressive gait instability accompanied by vertigo and nausea. Detailed neurologic examination revealed central nystagmus, bilateral dysdiadochokinesia, distal pallhypesthesia affecting the lower extremities, bilateral dysmetria on finger-nose-test, an ataxic gait and a positive Romberg test. Although ischemia, hemorrhage, malignancy as well as other morphological abnormalities of the cerebellum and cerebrum were immediately ruled out by CT and MRI of the head, the patient’s neurological symptoms deteriorated, necessitating the use of a walking aid.

In February, left-sided level II neck dissection took place and histologic work-up of the suspicious lymph nodes confirmed locoregional recurrence of the previously described spindle cell carcinoma. A short-term neurologic reevaluation confirmed persistence of cerebellar symptoms. In order to rule out infectious causes and autoimmune processes, serologic testing for syphilis and Lyme disease were done, but results were unremarkable. In addition, two sequential lumbar punctures were performed, cerebrospinal fluid analysis revealed cell counts (erythrocytes: 3/μl and 12/μl; monocytes: few; lymphocytes: few) and serum chemistry (total protein: 48 mg/dl and 44 mg/dl; glucose: 56 mg/dl and 57 mg/dl; lactate: 1.7 mmol/l and 1.8 mmol/l) within normal limits in the absence of malignant cells. Polymerase chain reaction and serology testing for commonly tested viruses (Herpes simplex virus, Varicella zoster virus, Ebstein-Barr virus, Cytomegaly virus, Tick-borne encephalitis virus, Enterovirus), protozoa (Toxoplasma gondii) and bacteria (Listeria, Borrelia) was negative. Magnetic resonance imaging of the spine was performed but did not show any morphological abnormalities either. Subsequently, the patient’s serum was analyzed for onconeural antibodies in order to investigate the possible role of a paraneoplastic syndrome in this case. The presence of anti-Hu antibodies was demonstrated by a tissue based assay for intracellular antigens, using an avidin-biotin peroxidase technique on frozen sections of rat cerebellum (antibody titer 1:2000; Fig. 3a/b) and confirmed by a recombinant immunoblot (ravo Diagnostika GmbH, Freiburg, Germany). Nerve conduction testing revealed the presence of demyelinating as well as axonal polyneuropathy of the lower extremities, affecting motor and sensory nerves. With regard to all findings, the patient’s neurological presentation was classified as paraneoplastic cerebellar degeneration. In the absence of other potential causative neurotoxic factors in the patient’s history, the polyneuropathy documented in parallel was also considered of paraneoplastic origin in association with detectable anti-Hu antibodies in the patient’s serum.

**Fig. 3:**
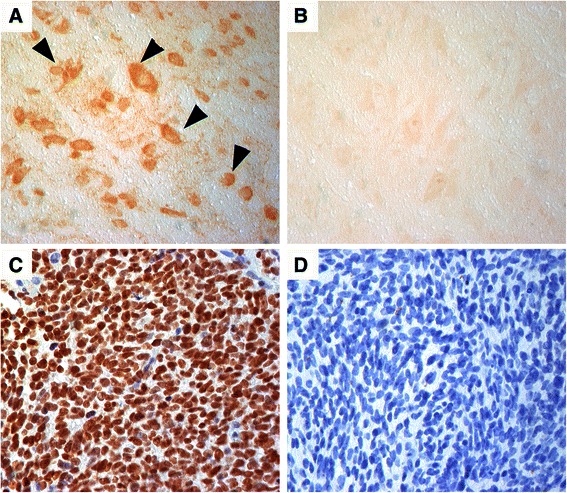
Immunohistochemistry on Rat Cerebellum and Primary Tumor Biopsy. Anti-Hu antibodies in the patient’s serum show an intensive labeling of nuclei and cytoplasm of brain stem neurons (arrowheads) (**a**). Serum of a healthy individual remains negative (**b**). Biopsy of the primary tumor reveals strong expression of Hu-antigen in the majority of tumor cells (**c**; biotinylated anti-Hu IgG). Staining with a biotinylated control IgG remains negative (**d**). Magnification: x400

Adjuvant concomitant chemoradiotherapy of the left neck with 64.6 Gy was initiated and the patient received two cycles of platinum-based chemotherapy at 21-day intervals starting in March 2010. In the light of the patient’s documented neuropathy, carboplatin AUC (area under the curve) 5 was preferred over cisplatin for concomitant use with radiotherapy due to its lower potential for peripheral neurotoxicity.

Complete remission was confirmed after multimodal cancer treatment and structural cerebellar and cerebral abnormalities were again ruled out by MRI of the head. After several months, the patient’s neurological symptoms, especially ataxia, had reached a plateau and she maintained her ambulatory ability by the use of a wheeled walker. On a regular basis, close interdisciplinary follow-up visits at the oncology and otorhinolaryngology department took place. Our patient remained in complete remission for the following 3 years without deterioration of ataxic gait or vertigo. Follow-up study of serum only revealed a slight decline of anti-Hu antibody titer (1:500). Immunohistochemical analysis on formalin-fixed and paraffin-embedded biopsy material of the primary tumor and the lymph node metastasis (not shown) with biotinylated anti-Hu IgG (obtained from an anti-Hu positive serum) showed strong nuclear expression of the Hu-antigen in the majority of tumor cells (Fig. 3c, d). However, in comparison to the primary tumor, the lymph node metastasis contained a substantial fraction dedifferentiated cells characterized by enlarged nuclei and enlarged cytoplasm.

As the patient’s neurological complaints remained stable for more than 3 years after effective anticancer therapy and considering the unsatisfying treatment responses described in the literature, we decided against immunosuppressive therapy or plasmapheresis.

## Discussion & conclusion

We present the first case of a patient with an underlying spindle cell carcinoma, formerly called carcinosarcoma, of the nasal cavity and ethmoid sinus who developed PCD associated with anti-Hu antibodies [10]. The majority of patients with PCD present with gynecological and breast cancer (anti-Yo or anti-Ri) as well as with SCLC (anti-Hu) [3]. In contrast, PNS are rarely observed in patients with head and neck cancer. Only two cases of PCD are reported, one laryngeal carcinoma without onconeural antibodies and one squamous cell carcinoma of the tongue with anti-CV2 antibodies [6, 7]. The spindle cell carcinoma of our patient (primary tumor and lymph node metastasis) showed strong immunoreactivity of the Hu-antigen, which confirms the association of anti-Hu antibodies and PCD associated with this type of tumor. Although the Hu-antigen was already expressed in the primary tumor, our patient’s neurological symptoms only started 1 year later at the time of tumor recurrence. In the majority of cases the PNS precedes the diagnosis of the underlying malignancy, however, it is not unusual that the neurological symptoms occur later during tumor recurrence and might be explained by altered antigen-presentation in the course of tumor dedifferentiation [3].

In most patients suffering from PCD, prognosis is determined by the outcome of their neurological syndrome rather than by the underlying malignancy. A retrospective analysis by Shams’ili et al. demonstrated poor neurological outcome and overall survival of patients with PCD, especially for those with detectable anti-Hu antibodies with a median survival of 7 months [3]. Antitumor therapy is still the gold standard for treatment of the neurological disorder while therapeutic approaches with immunoglobulins, cytotoxic agents, steroids or plasmapheresis aiming at stabilization or improvement of neurological symptoms proved to be of limited success [3, 11–13]. In contrast to the previously described common course of disease, our patient’s neurological symptoms stabilized after successful multimodal cancer therapy and she remained ambulatory with a walking aid for more than 3 years after development of PCD. It is noteworthy that the anti-Hu antibody titer in the patient’s serum had not significantly changed despite of complete remission for 3 years and stabilization of neurological symptoms. We also point out that results of nerve conduction testing were consistent with the presence of a mixed polyneuropathy, which might have contributed to the patient’s walking difficulties aside from cerebellar ataxia. In this respect, an association between non-cerebellar neurological manifestations and anti-Hu antibodies in patients with PCD has been observed [3]. Furthermore, in a retrospective analysis Camdessanché et al. describe paraneoplastic peripheral neuropathies in patients with positive anti-Hu antibodies in the serum [14].

Our approach in case of onset of new neurological symptoms associated with a known underlying malignancy (e.g. in head & neck cancer) includes obtaining detailed medical history, a thorough neurologic examination and neuroimaging (preferably MRI), and concomitant onconeural antibody screening in the patients’ sera [8]. Onconeural antibodies are most likely an epiphenomenon and T-cells lead to destruction of neurons and cell death. This is also reflected by the constant antibody-titer in our patient, despite of clinical stabilization and tumor remission. However, the antibodies serve as excellent biomarker in the diagnosis of a PNS. Detection of antineuronal antibodies is minimally invasive and helps to distinguish between autoimmune nature and treatment related side effects as putative cause of neurological disease. The diagnostic value of onconeural antibodies is characterized by a high specificity, whereas sensitivity is low to moderate meaning that PNS can occur without antibodies and a negative test result does not rule out presence of PNS [15]. Absence or presence of onconeural antibodies does not influence therapy of PNS, which is treatment of the underlying malignancy, instead, detection of these antibodies combined with consideration of the individual neurological syndrome is of prognostic value as overall survival is often limited by neurological outcome [3].

In summary, our case has several practical implications. First, in patients with head and neck tumors who develop neurological symptoms, a PNS should be considered as differential diagnosis. Second, despite of usually limited survival of patients with PNS associated with onconeural antibodies, long-lasting stabilization of neurological symptoms can be achieved by effective multimodal therapy of the underlying malignancy. Third, extensive screening of onconeural antibodies is important in order to establish/confirm the diagnosis of PNS and to evaluate prognosis.

## Consent

Written informed consent was obtained from the patient for publication of this case report and any accompanying images. A copy of the written consent is available for review by the Editor-in-Chief of this journal.

## Abbreviations

18F-FDG PET/CT: 18F-fluorodeoxyglucose positron emission tomography/computed tomography
AUC: area under the curve
Gy: gray
HE: Hematoxylin and Eosin
MRI: magnetic resonance imaging
PCD: paraneoplastic cerebellar degeneration
PNS: paraneoplastic neurological syndrome
SCLC: small cell lung carcinoma

## References

[1] Posner JB, Furneaux HM (1990). Paraneoplastic syndromes. Res Publ Assoc Res Nerv Ment Dis.

[2] Elrington GM, Murray NM, Spiro SG, Newsom-Davis J (1991). Neurological paraneoplastic syndromes in patients with small cell lung cancer. A prospective survey of 150 patients. J Neurol Neurosurg Psychiatry.

[3] Shams’ili S, Grefkens J, de Leeuw B, van den Bent M, Hooijkaas H, van der Holt B (2003). Paraneoplastic cerebellar degeneration associated with antineuronal antibodies: analysis of 50 patients. Brain.

[4] Minotti AM, Kountakis SE, Stiernberg CM (1994). Paraneoplastic syndromes in patients with head and neck cancer. Am J Otolaryngol.

[5] Baijens LW, Manni JJ (2006). Paraneoplastic syndromes in patients with primary malignancies of the head and neck. Four cases and a review of the literature. Eur Arch Otorhinolaryngol.

[6] Saloustros E, Zaganas I, Mavridis M, Vamvakas L, Plaitakis A, Georgoulias V (2010). Anti-CV2 associated cerebellar degeneration after complete response to chemoradiation of head and neck carcinoma. J Neuro-Oncol.

[7] Garcia FJ, Blazquez JA, Perez-Moro E, Martinez S (1998). [Paraneoplastic ataxia due to laryngeal carcinoma]. Acta Otorrinolaringol Esp.

[8] Graus F, Delattre JY, Antoine JC, Dalmau J, Giometto B, Grisold W (2004). Recommended diagnostic criteria for paraneoplastic neurological syndromes. J Neurol Neurosurg Psychiatry.

[9] Rojas I, Graus F, Keime-Guibert F, Rene R, Delattre JY, Ramon JM (2000). Long-term clinical outcome of paraneoplastic cerebellar degeneration and anti-Yo antibodies. Neurology.

[10] Zarbo RJ, Crissman JD, Venkat H, Weiss MA (1986). Spindle-cell carcinoma of the upper aerodigestive tract mucosa. An immunohistologic and ultrastructural study of 18 biphasic tumors and comparison with seven monophasic spindle-cell tumors. Am J Surg Pathol.

[11] Uchuya M, Graus F, Vega F, Rene R, Delattre JY (1996). Intravenous immunoglobulin treatment in paraneoplastic neurological syndromes with antineuronal autoantibodies. J Neurol Neurosurg Psychiatry.

[12] Keime-Guibert F, Graus F, Fleury A, Rene R, Honnorat J, Broet P (2000). Treatment of paraneoplastic neurological syndromes with antineuronal antibodies (Anti-Hu, anti-Yo) with a combination of immunoglobulins, cyclophosphamide, and methylprednisolone. J Neurol Neurosurg Psychiatry.

[13] David YB, Warner E, Levitan M, Sutton DM, Malkin MG, Dalmau JO (1996). Autoimmune paraneoplastic cerebellar degeneration in ovarian carcinoma patients treated with plasmapheresis and immunoglobulin. A case report. Cancer.

[14] Camdessanche JP, Antoine JC, Honnorat J, Vial C, Petiot P, Convers P (2002). Paraneoplastic peripheral neuropathy associated with anti-Hu antibodies. A clinical and electrophysiological study of 20 patients. Brain.

[15] Moll JW, Henzen-Logmans SC, Splinter TA, van der Burg ME, Vecht CJ (1990). Diagnostic value of anti-neuronal antibodies for paraneoplastic disorders of the nervous system. J Neurol Neurosurg Psychiatry.

